# American Dental Convention

**Published:** 1878-10

**Authors:** 


### AMERICAN DENTAL CONVENTION.

This body held a business meeting at Niagara Falls, Aug. 8th, and elected the following officers for the coming year:

President, Dr. A. C. Marvin, of Brooklyn, N. Y. Vice-president, J. R. Walker, of New Orleans, La. Secretary, Ambler Tees, Philadelphia. Treasurer, J. G. Ambler, New York. Saratoga was selected as the place for the next annual meeting, second Tuesday of August, 1879.

### SOUTHERN DENTAL ASSOCIATION.

The annual meeting of the *Southern Dental Association* was held at Niagara Falls, August 5th.

Owing to various circumstances, some of which at least could not have been anticipated, but two brief sessions were held which were wholly of a business character.

The officers were elected for the ensuing year, viz.: President, Prof. F. J. S. Gergas, Baltimore, Md.; First vice-president, L. D. Carpenter, Atlanta, Ga.; Second vice-president, J. R. Walker, New Orleans, La.; Third vice-president, J. G.

Wait, Richmond, Va.; Cor. Sec., A. C. Ford, Atlanta, Ga.; Treasurer, H. A. Laurence, Athens, Ga.; Executive Committee, W. C. Wardlow, Augusta, Ga.; G. N. Winkler, Augusta, Ga.; G. W. H. Whitaker, Sandersville, Ga.

The next, the eleventh annual meeting will be held at Augusta, Ga., on Tuesday, July eighth, 1879.

The aim and hope is to make this one of the best meetings ever held by this body, and for this object every member should put forth his best effort.

We trust it will be largely attended, and be made an interesting and profitable occasion.

